# *KIF5B-RET* Fusion gene may coincide oncogenic mutations of *EGFR* or *KRAS* gene in lung adenocarcinomas

**DOI:** 10.1186/s13000-015-0368-z

**Published:** 2015-08-14

**Authors:** Jeong-Oh Kim, Jieun Lee, Jung-Young Shin, Ji-Eun Oh, Chan-Kwon Jung, Jae Kil Park, Sook-Whan Sung, Sang-Ju Bae, Hyun-Jung Min, Dowon Kim, Jae Yong Park, Jin-Hyoung Kang

**Affiliations:** Laboratory of Medical Oncology, Reaserch Institutes of Medical Science, The Catholic University of Korea, 222 Banpo-daero Seocho-gu, Seoul, 137-701 Republic of Korea; Department of Medical Oncology, Seoul St. Mary’s Hospital, The Catholic University of Korea, 222 Banpo-daero Seocho-gu, Seoul, Republic of Korea; Division of Pathology, Seoul St.Mary’s hospital, The Catholic University of Korea, 222 Banpo-daero Seocho-gu, Seoul, Republic of Korea; Department of Thoracic and Cardiovascular Surgery, Seoul St.Mary’s hospital, The Catholic University of Korea, 222 Banpo-daero Seocho-gu, Seoul, Republic of Korea; Mirax Ltd, 334-1 Jangan-gu Suwon-si, Gyeonggi-do, Republic of Korea; Panagene Inc, 816 Tamnip-dong Yuseong-gu, Daejeon, Republic of Korea; Department of Internal Medicine, Kyungpook National University School of Medicine, 130 Dongdeok-ro Jung-gu, Daegu, Republic of Korea

**Keywords:** Lung adenocarcinoma, *KIF5B-RET* fusion gene, Fluorescence in situ hybridization

## Abstract

**Background:**

The *KIF5B-RET* rearrangement is detected with the frequency of 1 ~ 2 % in ‘triple marker’-negative lung adenocarcinomas, i.e., *EGFR, KRAS* and *EML4-ALK* wild type. These mutational changes are known to be mutually exclusive, but the co-existence of *ALK* rearrangement with activating mutations of *EGFR* is rarely found.

**Methods:**

We examined the *KIF5B-RET* fusion gene in frozen tissues from 154 surgically resected lung tumors using RT-PCR with direct sequencing and the mutation status of *EGFR* and *KRAS* genes using PNA clamping. We tested *KIF5B-RET* translocation in Formalin Fixed Paraffin Embedded using fluorescence in situ hybridization. We also measured RET mRNA and protein expression by RT-PCR and immunohistochemistry, respectively.

**Results:**

The existence of *KIF5B-RET* fusion gene was identified in 9 patients. The mean age was 67.2 and M: F ratio 4:5. Of 9 patients, 3 patients harbored wild type of *EGFR* and *KRAS* gene. However, *KIF5B-RET* fusion gene coincided with *EGFR* or *KRAS* mutation in 6 patients. These six pts were also positive for both RET break-apart probes (23.9 %) and *KIF5B-RET* fusion (44.4 %). However, there were no correlations between RET mRNA and protein expression in the KIF5B-RET-positive patients. The median disease free survival and overall survival were 23.9 months and 29.5 months, respectively.

**Conclusions:**

Taken together, our data suggest one-step screening platform for KIF5B-RET as well as *EGFR*, *K-RAS*, *ALK* oncogenic mutations be necessary for lung adenocarcinoma patients because *EGFR* or *KRAS* mutation are not infrequently found in *KIF5B-RET*-positive patients.

## Background

Over the past 10 years, great advances in molecular biology have enabled the discovery of various driver mutations of lung adenocarcinomas, including the epidermal growth factor receptor *(EGFR), ALK*, *AKT1, BRAF, HER2, KRAS, MET, PIK3CA, RET*, *ROS1*. The proliferation of tumor cells that originates from a single oncogene mutation is termed as an ‘oncogene addiction’. Specific blocking of an oncogene effectively inhibits the growth of tumor cells, leading to the development of targeted drugs in oncology [1–3].

In 2012, we first identified the existence of the Kinesin family member 5B (*KIF5B)-RET* fusion gene in a young male patient with lung adenocarcinoma using whole-genome and transcriptome sequencing analysis. This gene rearrangement, involving the fusion of exon 16 from *KIF5B* and exon 12 from *RET* can strongly influence oncogenesis [4]. The *KIF5B-RET* fusion gene is rarely found in Asians or never smokers, and is mutually exclusive to other oncogenic mutations including those in the *EGFR, KRAS*, *BRAF*, and *ERBB2* gene, or *EML4-ALK* gene. The *KIF5B-RET* fusion gene has been reported to occur in 1–2 % of lung adenocarcinomas; however, adenocarcinomas of other anatomical sites lack this mutation. Moreover, the correlation of *KIF5B-RET* fusion gene with smoking is still ambiguous [5].

The transplantation of the K15:R12 variant of *KIF5B-RET* fusion gene–transformed cells into nude mice resulted in the development of the tumor *in vivo*, indicating the fusion gene’s oncogenic potential [6,7]. In the ongoing NCT001639508 study, cabozantinib demonstrated disease control activity for patients harboring *RET* gene rearrangements, including the *KIF5B-RET* fusion gene [8]. These encouraging preliminary results suggest that the *RET* fusion gene may be a promising druggable target [8,9]. Although the aforementioned driver mutations were accepted to be mutually exclusive, several recent studies have reported the co-existence of *ALK* rearrangement with *EGFR* or *KRAS* mutations. Moreover, other investigators have shown that the sequential administration of the EGFR tyrosine kinase inhibitor (TKI) and ALK inhibitors could induce stable disease for the patients harboring concomitant *EGFR* mutation and *ALK* rearrangement [10]. These results imply that patients harboring concomitant oncogenic driver mutations may show different biologic behaviors and clinical outcomes to the targeted therapy.

In this study, we examined the frequency of the *KIF5B-RET* fusion gene in Korean NSCLC patients and whether this gene rearrangement coincides with other oncogenic drivers.

## Methods

### Patients

Between February 2009 and June 2013, we enrolled patients who were diagnosed with pulmonary adenocarcinomas at Seoul St. Mary’s Hospital and Korea Lung Tissue Bank of the Infrastructure Project of Basic Science of the Ministry of Education, Science, and Technology. All patients received surgical pulmonary resection that was pathologically confirmed as an adenocarcinoma by a senior pathologist. All tumor samples were immediately frozen after surgical resection, and stored at −80 °C until the experiment. Remaining tumor samples were stored as formalin-fixed paraffin-embedded (FFPE) tissues.

Written informed consent was obtained from all participants, and this study was approved by the Institutional Review Board of Seoul St Mary’s Hospital (approval No. KC13SISI0040) and Guro Hospital, Korea University (approval No. KU Guro Gene Bank.2013-015).

### Reverse transcription polymerase chain reaction (RT-PCR) for the KIF5B-RET fusion gene and RET mRNA expression

Total RNA were extracted from frozen tissues as per manufacture’s protocols using ZR-Duet DNA/RNA Miniprep kit (Zymoresearch, Irvine, CA). RNA concentrations were determined using the Nano Drop ND-1000 spectrophotometer (Nano Drop Technologies Inc., Rockland, DE). After the synthesis of cDNA using the Maxime RT Premix (INTRON Biotechnology Inc, Korea), we designed as previously reported [4]. The sequences for the *RET* gene’s kinase domain were used to design the forward primer: 5′-GAA GGC GAA TTT GGA AAA GT-3′, and the reverse primer: 5′-ATACTG CAT CCC CTG TGA GA-3′. The reaction conditions were as follows: 40 cycles of 95 °C for 10 sec, 58 °C for 40 sec and 72 °C for 7 sec, with a final extension for 10 min at 72 °C. Glyceraldehyde 3-phosphate dehydrogenase (GAPDH) was used as internal reference gene.

### PNA Clamp^TM^ EGFR and KRAS mutation analysis

Genomic DNA was determined using the Nano Drop ND-1000 Spectrophotometer. Mutational analysis for *EGFR* and *KRAS* was performed using the CFX96 Real-Time PCR Detection System (Bio-Rad, Philadelphia, PA) with the PNA clamp^TM^*EGFR* and *KRAS* mutation detection kit (Panagene, Inc., Daejeon, Korea). All reactions performed as previously reported [2].

### Analysis of the *KIF5B-RET* fusion gene using FISH

To detect the *KIF5B-RET* fusion gene in tumor tissues using FISH, 4 μm-thick FFPE sections were used; FFPE slides were deparaffinized and rehydrated, and were pretreated with citrate buffer (Cellay Inc., Cambridge, MA) for 15 minutes at 96–98 °C, and then immersed in pepsin solution for 10 minutes at 37 °C. The FISH probe was denatured for 5 min at 80 °C, subsequently the slides were incubated for 20 hours at 37 °C overnight.

The ZytoLight ® SPEC RET Dual Color Break Apart Probe set (Zytovision Inc. Bremerhaven, Germany), KIF5B-RET SY Translocation FISH probe (Abnova, Taipei, Taiwan) and FISH detection kit were applied to the slides. After the post-hybridization washing, 4’, 6-diamidino-2-phenylindole were used for counter staining. The stained slides were assessed using a Carl Zeiss microscope with the tissue FISH analysis module using the Metafer software (MetaSystems, GmbH Altlussheim, Germany).

### Interpretation of immunohistochemical staining

Immunohistochemical staining was performed on 4 μm thick FFPE tumor tissue sections. The slides were deparaffinized, and antigen retrieval was performed in a steam cooker for 15 minutes in citrate buffer (0.01 M, pH 6.0). The sections were incubated overnight at 4 °C with primary antibodies for anti-RET (Rabbit monoclonal, 1:500 dilution, Epitomics, California, USA) and anti-ALK (Rabbit monoclonal, 1:250 dilution, Cell Signaling Technology, Danvers, MA, USA). After washing, signal was amplified by incubating the section with secondary antibody (Polink-2 plus HRP Rabbit DAB kit; GBI,Mukilteo,WA, USA), for 10 minutes. The following rabbit monoclonal antibodies were used for immunohistochemical staining. Finally, the reaction was visualized with a 3,3’-diaminobenzidine as the chromogens. Slides were counterstained with hematoxylin before mounting. The levels of RET and ALK expression were scored on the basis of the staining intensity of tumor cells, and the relative proportion of positively stained cells among total tumor cells. The tumor cell staining intensity was graded as follows: 0, absent; 1, weak (light brown); 2, moderate (brown); and 3, strong (dark brown).

### Statistical analysis

Overall survival was calculated from the date of clinical diagnosis of lung adenocarcinoma to the death of patient, or patient’s last follow-up date. Disease-free survival was calculated from the date of surgical resection to the date of disease recurrence, which was confirmed by imaging studies including chest CT scans. The statistical correlations between the results of immunohistochemistry for RET and FISH analysis for the *KIF5B-RET* gene rearrangement was analyzed using Student’s t tests.

The frequency of *KIF5B-RET* fusion and *RET* mRNA expression was compared with paired t-test. All statistical analyses were performed with SPSS (ver. 18.0; SPSS, Chicago, IL, USA).

## Results

### Baseline characteristic

Among 154 patients, 83 patients were male (53.9 %), with no gender predominance. The median age was 62 years (range, 25–85 years). One hundred and fifteen patients (74.4 %) had an eastern cooperative oncology group performance status of 0, 96 (62.3 %) were classified with stage I disease, and 45 patients (29.3 %) with stage II or III. Ninety-four patients (61.0 %) had moderately- differentiated adenocarcinomas (Table 1).

**Table 1:** Basic Characteristics of Patient’s

Patient’s Characteristics	No. (%)
No. of patients	154
Median age(range)	62 (25-85)
Sex	
Male	83(53.9%)
Female	71(46.1%)
Smoking	
Never smoker	80(51.9%)
Ever smoker	74(48.1%)
ECOG	
0	115(74.4%)
1	39(25.6%)
Initial stage	
I	96(62.3%)
II	24(15.6%)
III	21(13.7%)
IV	13(8.4%)
Differentiation	
Well differentiated	43(27.9%)
Moderate differentiated	94(61.0%)
Poorly differentiated	17(11.1%)

### Distribution of the KIF5B-RET fusion gene, and EGFR and KRAS mutations

**Fig. 1:**
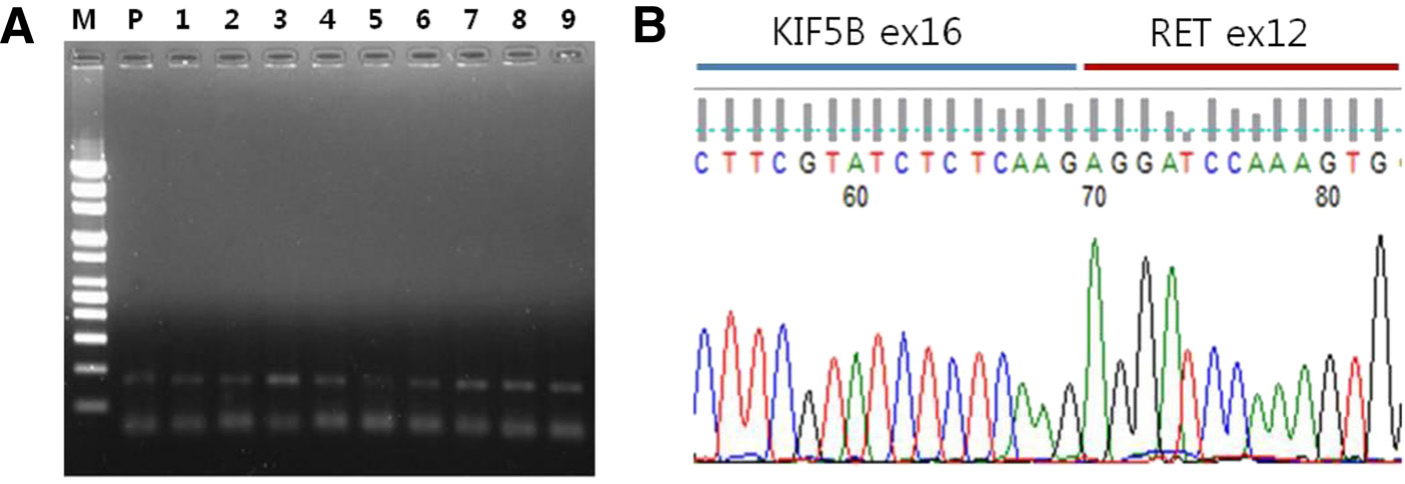
*KIF5B-RET* fusion gene of RT-PCR and direct sequencing. **a** RT-PCR was analyzed using cDNA from 154 NSCLC patients. Samples observed the amplified *KIF5B-RET* fusion gene in 9 patients. **b** We confirmed that the *KIF5B-RET* varient 16/12 by direct sequencing in all patients. Lane 1, size marker (M); lane 2, positive control (P); lane 3–12, number of patients

**Fig. 2:**
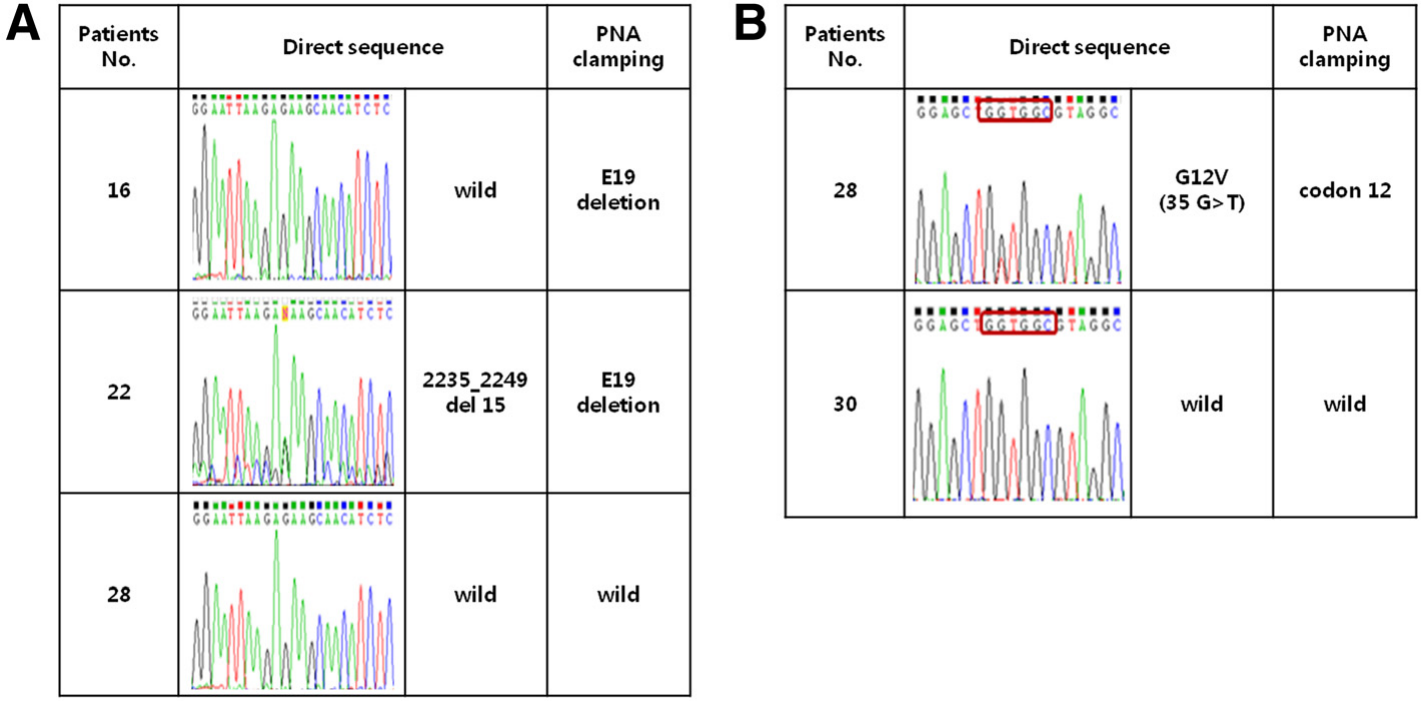
*EGFR* and *KRAS* gene mutations in patients. *EGFR* (**a**) and *KRAS* (**b**) mutations were identified using the PNA clamping and direct sequencing method for 9 patients. PNA clamping analysis revealed similar results as those obtained from direct sequencing, one patient exhibited *EGFR* 19del on PNA clamping, but a weak mutant peak on direct sequencing

The expression of the *KIF5B-RET* fusion gene was analyzed in 154 archival tumor tissues. The mRNA was extracted from fresh frozen tumor tissue, and amplified by RT-PCR. The primers were designed based on the *KIF5B* exon 15, 16, and *RET* exon 12 (K15, 16, R12). We observed the amplified *KIF5B-RET* fusion gene in 9 patients (5.8 %) (Fig. 1a). Next, we confirmed the *KIF5B-RET* variant 16/12 by direct sequencing in all patients (Fig. 1b). In addition, we conducted direct sequencing and PNA clamping to examine whether the patients harbored *EGFR* or *KRAS* mutations. PNA clamping analysis revealed that 5 of 9 patients harbored an activating *EGFR* mutation, 2 had a microdeletion in exon 19, and 3 harbored missense mutations in exon 21 (L858R). For 5 patients, the *EGFR* mutation status was confirmed with direct sequencing; equivalent results were obtained, except for one patient with a microdeletion in exon 19, where a weak mutation peak was observed. Among the 9 patients, 1 patient (11.1 %) harbored a KRAS codon 12 mutation, which was analyzed by direct sequencing and PNA clamping (Fig. 2). The remaining 3 patients (33.3 %) were positive for the *KIF5B-RET* fusion gene exclusively, and harbored no *EGFR* and *KRAS* mutations, or *ALK* rearrangement.

### The detection of KIF5B-RET fusion gene by FISH

**Fig. 3:**
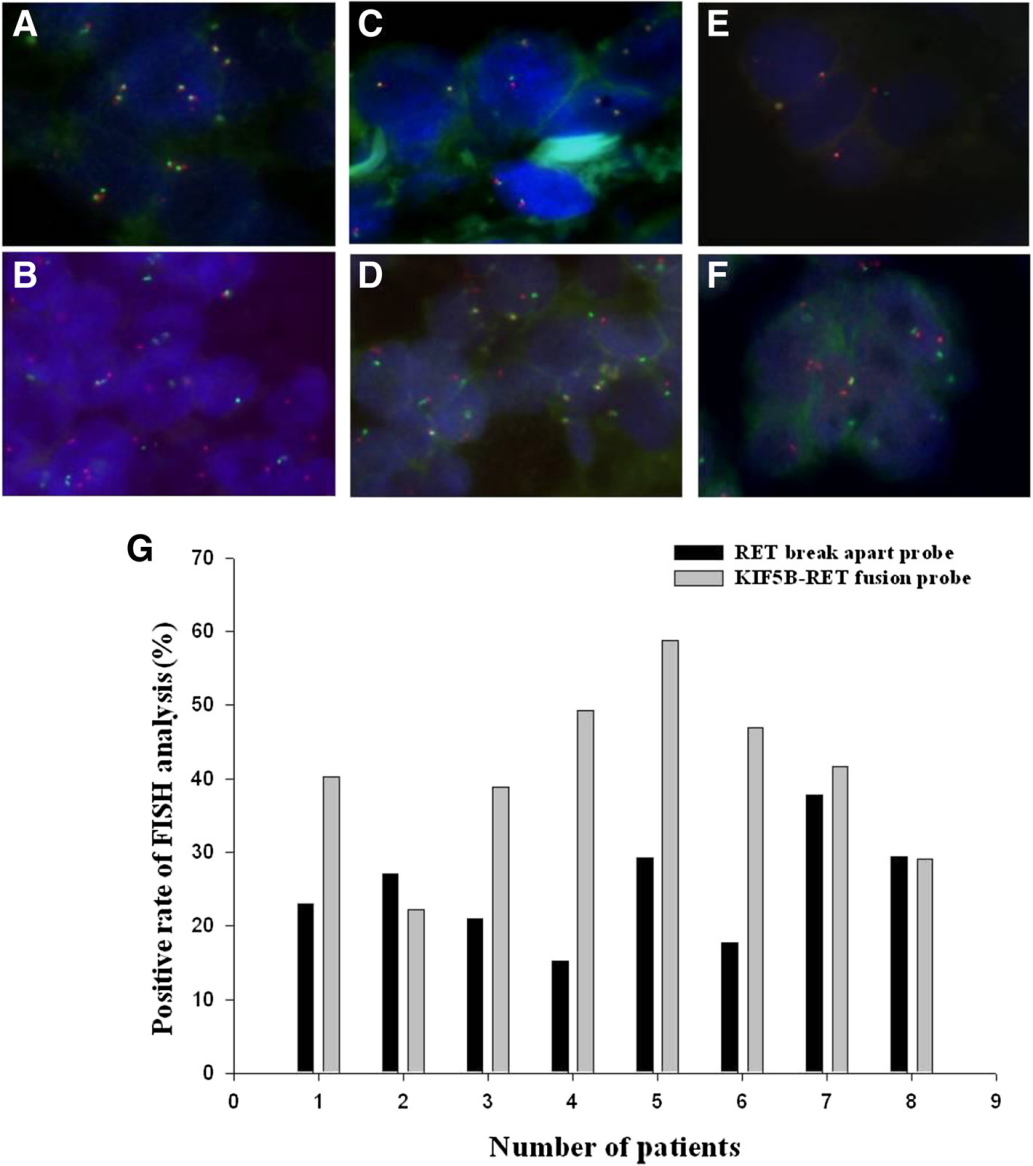
FISH analysis for *RET* break apart probe (**a**, **c**, **e**) and *KIF5B-RET* fusion probe (**b**, **d**, **f**). **a** and **b** show two types of FISH analysis for both wild type of *EGFR* and *KRAS* mutation status. **c** and **d** show FISH analysis for E19del mutation and wild type of *KRAS* mutation. **e** and **f** depict FISH analysis for wild type of *EGFR* mutation and codon12 of *KRAS* mutation. **g** Comparing the frequency of *RET* break apart probe and *KIF5B-RET* fusion probe

FISH analysis was performed to confirm the existence of the *KIF5B-RET* fusion gene at the DNA level in 9 samples with *KIF5B-RET* mRNA, as identified by RT-PCR. We counted 100 interphase nuclei within each tumor specimen in 100 X high power fields under fluorescence microscopy. Tumor tissue with > 15 % split red and green signal in 100 interphase nuclei was defined as positive for the *RET* fusion gene (Fig. 3). In our study, the average frequency of the RET break-apart probes 23.9 % (range, 14.8–37.8) and *KIF5B-RET* fusion 44.4 % (range, 22.2-72.4)in 9 tumor tissues (Fig. 3G). The average FISH split and fusion signal for RT-PCR negative cases were 4 % (range, 2.1-10) and 18.8 % (range,14.4-23.9), respectively.

### The correlation between KIF5B-RET fusion gene and RET mRNA expression, and ALK protein expression by immunohistochemistry

**Fig. 4:**
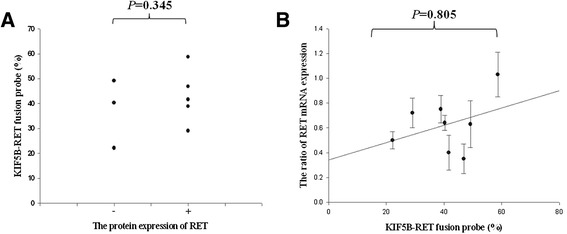
The expression of *RET* mRNA and protein in *KIF5B-RET* fusion gene patients. **a** The patients having RET protein expression tended to be positive *KIF5B-RET* fusion gene by FISH, but with no statistical significance (*P* = 0.345). **b** FISH analysis and *RET* mRNA expression of the *KIF5B-RET* fusion gene was observed. There was no statistical association between *RET* mRNA and FISH (*P* = 0.805)

**Fig. 5:**
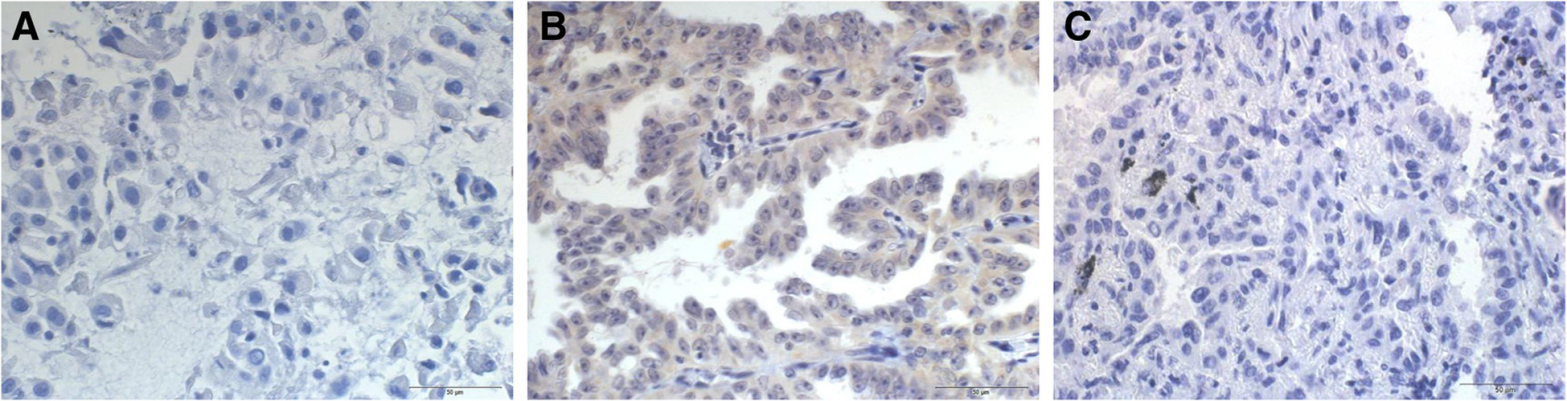
The protein expression of RET and ALK (D5F3) using immunohisto-chemistry. Specimens (**a**, **b**) show negative and positive of RET IHC with *RET rearrangement* status of FISH analysis. Specimens (**c**) show negative ALK IHC with *RET rearrangement* status (original magnification, ×400)

Total RNA was extracted from the fresh frozen tumor tissues, in which the presence of *KIF5B*-*RET* fusion gene was confirmed by both RT-PCR and FISH analysis. We analyzed the correlation between the frequencies of *KIF5B-RET* fusion by FISH and *RET* mRNA, or RET protein expression. In our analysis, the samples with RET protein expression tended to be positive for expression of the *KIF5B-RET* fusion gene by FISH, however the correlation was not statistically significant (*P* = 0.345) (Fig. 4a and Fig. 5b). Moreover, no statistical association could be determined between *RET* mRNA and expression of the *KIF5B-RET* fusion gene by FISH (*P* = 0.805) (Fig. 4b). Also, we analyzed ALK protein expression by immunohistochemical analysis using a monoclonal antibody specific for ALK fusion protein; no ALK expression could be detected in samples harboring the *KIF5B-RET* fusion gene (Fig. 5c).

### The association between the KIF5B-RET fusion gene and clinical variables

We reviewed the clinical progress of 9 patients harboring the *KIF5B-RET* fusion gene. The median age was 67.2 years (range 56–82 years); 4 patients were male. Five patients were never smokers. The median relapse-free survival duration was 23.9 months (range 2.63–110.8 months), and median overall survival was 29.5 months (range 2.6–114.5 months). All patients had histologically confirmed adenocarcinomas with acinar, lepidic, or papillary growth patterns. We detected the *KRAS* and *EGFR* mutation as well as the *KIF5B-RET* fusion gene in primary lung adenocarcinomas. We concluded that the lung tumor samples were based on the clinical history and the immunohistochemical data, which showed no expression of thyroid transcription factor (TTF-1) and napsin. In summary, 6 of 9 patients had either *EGFR* or *KRAS* mutations, and the remaining 3 patients were negative for both *EGFR* and *KRAS* mutations (Table 2).

**Table 2:** Clinical and genetic characteristics of patients carrying *KIF5B-RET* fusion gene

No	Clinical characteristics			Genetic characteristics						
	Sex/Age	Smoking	Histology	*KIF5B-RET* fusion gene		*EGFR* mutation	*KRAS* mutation	ALK rearrangement	OS^a^ (mo)	DFS^b^ (mo)
				Break apart probe (%)	Fusion probe (%)					
1	F/58	Never-smoker	Acinar	23.0	40.3	Wild	Wild	negative	8.7	7.33
2	M/82	current	Acinar	27.1	22.2	Wild	Wild	negative	114.5	110.8
3	M/63	Ex-smoker	Acinar	21.0	38.9	Wild	Wild	negative	53.4	15.23
4	F/71	Never-smoker	Acinar& lepidic	15.3	49.2	E19del	Wild	negative	9.6	9.63
5	M/56	Ex-smoker	Papillary	29.2	58.7	E19del	Wild	negative	11.2	10.8
6	F/57	Never-smoker	Acinar& lepidic	17.8	46.9	L858R or L861Q	Wild	negative	2.6	2.63
7	F/74	Never-smoker	Acinar& lepidic	37.8	41.7	L858R or L861Q	Wild	negative	5.9	5.9
K15	F/74	Never-smoker	Acinar	14.8	72.4	L858R	Wild	-	FU loss	FU loss
8	M/70	Ex-smoker	Papillary	29.4	29.1	Wild	Codon12	negative	30.1	29.37

^a^Overall survival; ^b^Disease free survival

## Discussion

The *KIF5B-RET* fusion gene is capable of inducing the abnormal proliferation and differentiation of tumor cells through the constitutional overexpression and activation of the *RET* proto-oncogene, ultimately leading to lung adenocarcinomas [4]. To date, the *EGFR* and *KRAS* gene mutations, and the *EML4-ALK* fusion gene have been identified as major oncogenic driver mutations of lung adenocarcinomas. However, the molecular etiology of ~40 % of adenocarcinomas remains to be discovered [3]. In the event that screening tests fail to detect driver mutations in advanced adenocarcinomas, palliative chemotherapy is the main treatment option.

Data from previous studies have consistently indicated that oncogenic driver mutations are mutually exclusive [3]. However, recent studies have shown that the *ALK* fusion gene was found may occur concomitantly with the *EGFR* mutation. Yang et al. reported that the frequency of concomitant *EGFR* mutation and *ALK* translocation was 1.3 % in NSCLC [11]. Upon administering a specific tyrosine kinase inhibitor (TKI), dissimilar responses were observed in the patients harboring concomitant genomic alterations as compared to patients with single genetic alteration. Recently, Hirai et al. presented a case exhibiting poor response after the administration of an *EGFR* TKI in a lung adenocarcinoma patient harboring both, the *KIF5B-RET* fusion gene and *EGFR* mutation [12]. In patients with concurrent *EGFR* mutation and *ALK* rearrangement, the tumor response to certain TKIs may be inferior to historical data, depending on which genetic alteration is the major oncogenic driver. This hypothesis may explain the intrinsic resistance to *EGFR* TKIs in patients harboring concomitant genetic alterations [11,12]. We investigated the incidence of the *KIF5B-RET* fusion gene in Korean NSCLC patients, and attempted to identify the cases in which this fusion gene occurred concurrently with *EGFR* or *KRAS* mutations. We discovered *KIF5B-RET* fusion genes in 9 (5.8 %) out of 154 patients with lung adenocarcinoma, at a higher frequency than was reported previously [13]. Most prior studies had investigated the frequency of the *KIF5B-RET* fusion gene in an enriched cohort that was confirmed as triple negative by screening for the *EGFR* and *K-RAS* mutations, and *EML/ALK* rearrangement [13,14].

Conversely, in the present study, we screened all patients for the *KIF5B-RET* fusion genes using RT-PCR, irrespective of the status of *EGFR* and *KRAS* mutations, or *EML/ALK* fusion genes. The frequency of the *KIF5B-RET* fusion gene in patients lacking genetic alterations including *EGFR*, *KRAS*, and *ALK* rearrangement was 1.9 %, similar to the results of the previous studies [5,15]. It is reasonable to assume that any discrepancies in the frequency of *KIF5B-RET* fusion gene may arise from dissimilarities in study populations. To date, seven variant isoforms of the *KIF5B-RET* fusion gene have been identified; K15; R12 is the most common variant, accounting for 60–70 % of *KIF5B-RET* gene variants [5,16]. In our study, we performed direct sequencing to verify the existence of variant K16; R12 and K15; R12, and then ascertained that K16; R12 was present in tumor tissues of all cases.

In addition, we carried out PNA clamping and direct sequencing to analyze whether the *EGFR* or *KRAS* mutation may coincide with *KIF5B-RET*-positive patients. One patient exhibited *EGFR* E19del on PNA clamping, but a weak mutant peak on direct sequencing [17]. We used next-generation sequencing to verify that PNA clamping was more sensitive and accurate. Currently, FISH is widely accepted to be the most useful tool for detection of structural genetic variations such as the *RET* rearrangement; it is a simpler, faster, and less expensive standard method, compared with whole-genome sequencing or transcriptome sequencing.

The results obtained from two types of FISH analysis were in agreement; as the cut-off level for positive cells in known potential *ALK* gene rearrangement was used, the frequencies of *RET* rearrangements in the two FISH analyses may be higher than the cutoff of the *ALK* gene analysis. In the case of *ALK*, the cut-off criteria was determined on the basis of an association study with the ALK inhibitor crizotinib [18]. We evaluated the RET protein expression induced by *KIF5B-RET* fusion by immunohistochemical analysis of paraffin-embedded tumor tissues with a RET C-peptide monoclonal antibody. Relatively weak staining intensity (1+) of RET protein was observed in most samples harboring the *KIF5B-RET* fusion gene as identified by RT-PCR and FISH analysis; however, the average intensity was higher than that of the patients with no *KIF5B-RET* fusion. A recent paper indicated that weak intensity of RET protein expression was observed in *KIF5B-RET* fusion-positive patients, which is in agreement with our observation [15]. In the present study, we observed a trend towards stronger intensity of RET protein expression concurrent with an increase in frequency of *KIF5B-RET* fusion gene (*P* = 0.345).

Conversely, no statistical significance was observed between the frequencies of *KIF5B-RET* fusion gene and *RET* mRNA expression (*P* = 0.805). These results imply that neither RET protein expression nor *RET* mRNA expression can successfully indicate the presence of the *KIF5B-RET* fusion. Structural alterations within the KIF5B-RET fusion protein may explain the lower reactivity to the conventional RET monoclonal antibody used in our study; thus, it is necessary to develop a specific antibody in order to assess the expression of KIF5B-RET fusion protein. Moreover, the correlations obtained with FISH analysis should be evaluated by measuring gene amplification, rather than mRNA expression of *KIF5B-RET* fusion gene, as shown by Go et al. [19].

In contrast to previous reports, our results indicate that the *KIF5B-RET* fusion gene may coincide with *EGFR* or *KRAS* mutations, albeit at a lower frequency, in lung adenocarcinomas. Further research is required to understand the functional molecular genetics of *KIF5B-RET* fusion gene, and to facilitate the development of a novel therapeutic drug targeting this fusion gene.

## Conclusions

Our study demonstrated that the *KIF5B-RET* fusion gene was associated with *EGFR or KRAS* mutations, albeit at a low frequency. Therefore, screening for the *EGFR* and *KRAS* mutations, and *ALK* and *KIF5B-RET* translocations should be considered during initial diagnosis of lung adenocarcinomas. Moreover, clinical studies that combine RET inhibitors and EGFR TKIs with other targeted drugs are warranted for the lung adenocarcinoma patients harboring concurrent *KIF5B-RET* fusion gene and *EGFR* or *KRAS* mutations.

## Abbreviations

*KIF5B-RET*: Kinesin family member 5B
EGFR TKI: Epidermal growth factor receptor tyrosine kinase inhibitor
FFPE: Formalin-fixed paraffin-embedded
RT-PCR: Reverse transcription polymerase chain reaction
FISH: Fluorescence in situ hybridization
GAPDH: Glyceraldehyde 3-phosphate dehydrogenase
